# Correction: ter Woerds et al. Endobronchial Ultrasound Staging During Navigation Bronchoscopy for Peripheral Pulmonary Nodules in the Real World: Which Patients Will Benefit? *Cancers* 2025, *17*, 1700

**DOI:** 10.3390/cancers17152450

**Published:** 2025-07-24

**Authors:** Desi K. M. ter Woerds, Roel L. J. Verhoeven, Ad F. T. M. Verhagen, Erik H. J. G. Aarntzen, Erik H. F. M. van der Heijden

**Affiliations:** 1 Department of Pulmonary Diseases, Radboud University Medical Center, 6525 GA Nijmegen, The Netherlands; desi.terwoerds@radboudumc.nl (D.K.M.t.W.);; 2 Department of Thoracic Surgery, Radboud University Medical Center, 6525 GA Nijmegen, The Netherlands; 3 Department of Medical Imaging, Radboud University Medical Center, 6525 GA Nijmegen, The Netherlands; 4 Department Nuclear Medicine and Molecular Imaging, University Medical Center Groningen, 9713 GZ Groningen, The Netherlands; 5 Department of Nuclear Medicine, Eberhard Karls University Tuebingen, 72074 Tuebingen, Germany

**Error in Table**

In the original publication [1], there was a mistake in Appendix B, Table A2, as published. The calculations concerning patients with a negative EBUS and a pN0 stage after surgery were incorrect. The previous version incorrectly assumed that all 88 patients underwent surgery within six weeks after navigation bronchoscopy. However, this was not the case. An additional specificity has now been calculated, and the negative predictive values (NPV) for this subgroup have been adjusted accordingly. The corrected Table A2 appears below. The authors state that the scientific conclusions are unaffected. This correction was approved by the Academic Editor. The original publication has also been updated.

## Figures and Tables

**Table A2:** The sensitivity, specificity, PPV, and NPV of EBUS in the cohort of lung cancer patients with cytologically confirmed N-status by EBUS or pN-status confirmed by surgery. ^a^ One false-positive lymph node (atypical cells in pathology, MDT decision cN1). ^b^ In two patients, only micrometastases were found in station 12, which is inaccessible by EBUS. Additional calculations were performed by excluding patients with nodal involvement in EBUS inaccessible regions. ^c^ In six patients, surgery was performed after more than 6 weeks due to comorbidities, neo-adjuvant treatment, or treatments of synchronous cancers. In this subgroup, pulmonary surgery was performed 47 to 136 days after the NB + EBUS, and they were, therefore, left out of this secondary analysis. Abbreviations: EBUS, Endobronchial Ultrasound; NB, Navigation Bronchoscopy; NNT, Number Needed to Treat; NPV, Negative Predictive Value; PPV, Positive Predictive value.

Accuracy of EBUS in Surgically Treated cN0 Lung Cancer Patients (See Main Manuscript for cTNM and pTNM Details, Median Tumor Size 17 mm)			
Lung cancer patient subgroup: NB + EBUS with surgical confirmation (*n* = 118)	Pathology N-status		
	pN+	pN−	Total
EBUS N+	12	1 ^a^	13
EBUS N−, all (accessible ^b^)	17 (15)	88	105
EBUS N−, surgery < 6w after NB (accessible ^b^) ^c^	12 (10)	61	
Total	29	89	118
Sensitivity (all)	12/(12 + 17)		41.4%
Sensitivity (EBUS accessible)	12/(12 + 15)		44.4%
Sensitivity (surgery < 6w after NB)	12/(12 + 12)		50.0%
Sensitivity (surgery < 6w after NB + EBUS accessible)	12/(12 + 10)		54.5%
Specificity (all)	88/(1 + 88)		98.9%
Specificity (surgery < 6w after NB)	61/(1 + 61)		98.4%
PPV	12/(12 + 1)		92.3%
NPV (all)	88/(17 + 88)		83.8%
NPV (EBUS accessible)	88/(15 + 88)		85.4%
NPV (surgery < 6w after NB)	61/(12 + 61)		83.6%
NPV (surgery < 6w after NB + EBUS accessible)	61/(10 + 61)		85.9%
Overall accuracy	(12 + 88)/118		84.7%
NNT overall			10
NNT iN+ subgroup			4
NNT iN− subgroup			∞
